# Maxillary Swing Approach for Removal of Palatal Carcinoma: A Modified Procedure

**DOI:** 10.1155/2018/5318207

**Published:** 2018-04-05

**Authors:** Tsutomu Nomura, Megumi Kishimoto, Hirohisa Iwaki, Atsushi Ochi, Seiji Kishimoto

**Affiliations:** ^1^Department of Otorhinolaryngology, Saitama Medical Center, Saitama, Japan; ^2^Department of Otorhinolaryngology, Kameda Medical Center, Kamogawa, Japan; ^3^Department of Head and Neck Surgery, Kameda Medical Center, Kamogawa, Japan

## Abstract

**Introduction:**

We report a modification of the maxillary swing approach to remove a palatal tumor while preserving the anterior alveolar area.

**Methods:**

Case report using clinical records.

**Results:**

The patient was a 54-year-old male. TNM grade was T4bN0M0, and invasion to the base of the pterygoid process was seen. Two courses of induction chemotherapy were administered prior to the operation. Because there was no evidence of anterior maxillary invasion, the maxillary swing approach was chosen. The left anterior maxilla was cut and swung laterally, preserving the blood supply. After removal of the palatal tumor, the maxilla was repositioned and the defect was restored with an anterior lateral thigh flap. Postoperative course was typical, and facial appearance, speech, and masticatory function were satisfactory.

**Conclusions:**

This technique is particularly useful for preserving appearance as well as speech and mastication.

## 1. Introduction

In surgical treatment for tumors invading to the pterygoid fossa, complete resection with adequate surgical margins is challenging because of the anatomic complexity of the maxillofacial region. To overcome this issue, Wei et al. [1] developed the maxillary swing (MS) approach to the nasopharyngeal area; this was later modified by Sumi et al. [2] into the partial maxillary swing approach. A further modified version of the technique was reported by Shimoda et al. [3]. Here, we used an anteriorly modified form of MS to preserve the anterior alveolar area while removing a palate tumor invading the pterygoid lesion.

## 2. Case Report

**Figure 1:**
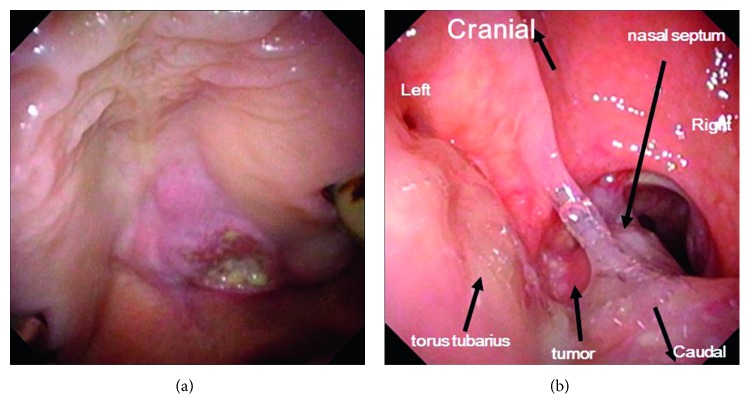
(a) Oral view of the palatal tumor. (b) Posterior nasal aperture view. Fiberscope was inserted from mouth and turned to posterior nasal aperture via uvula. Fiberscope tip was turned upside down, and posterior nasal aperture view was taken. Tumor can be seen in the left posterior nasal meatus area and is clearly invading the nasal septum. Right torus tubarius is intact.

**Figure 2:**
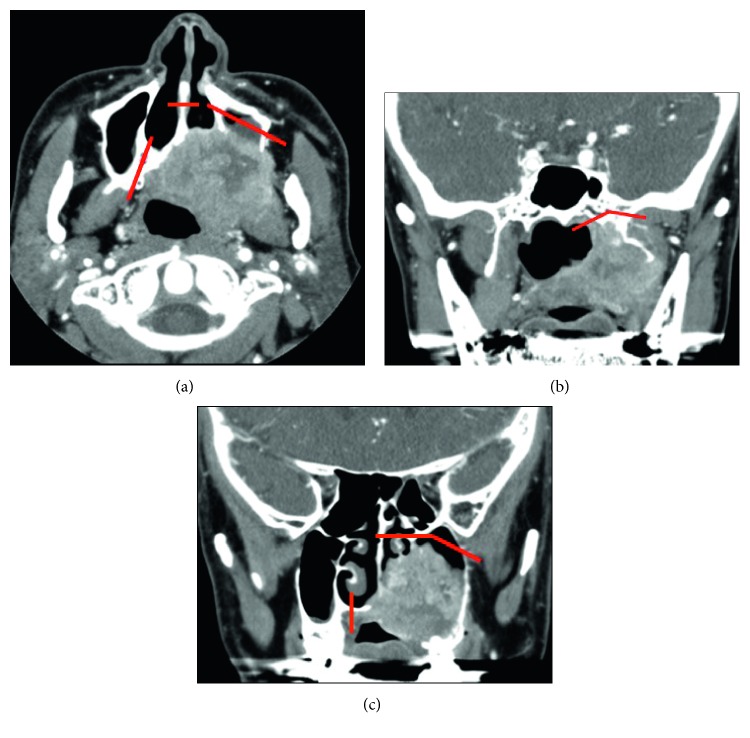
CT views. (a) Axial view: tumor invading the left posterior maxillary sinus but not the anterior area. (b) Coronal view: tumor invading the base of the pterygoid process. (c) Tumor not invading the orbital fossa or cranial bone. Red lines are dissection lines.

A 54-year-old man who had complained of ongoing pain in the palate was referred to our department with a diagnosis of a palatal squamous cell carcinoma. The stage T4bN0M0 tumor had formed an ulcer in the palate and invaded the posterior portion of the inferior nasal turbinate. The pharyngeal orifice of the auditory tube was intact (Figure 1). Coronal view CT showed that the tumor had invaded the base of the pterygoid process without cranial base invasion (Figure 2(b)). After two courses of induction chemotherapy, the tumor was found to have disappeared from this area.

**Figure 3:**
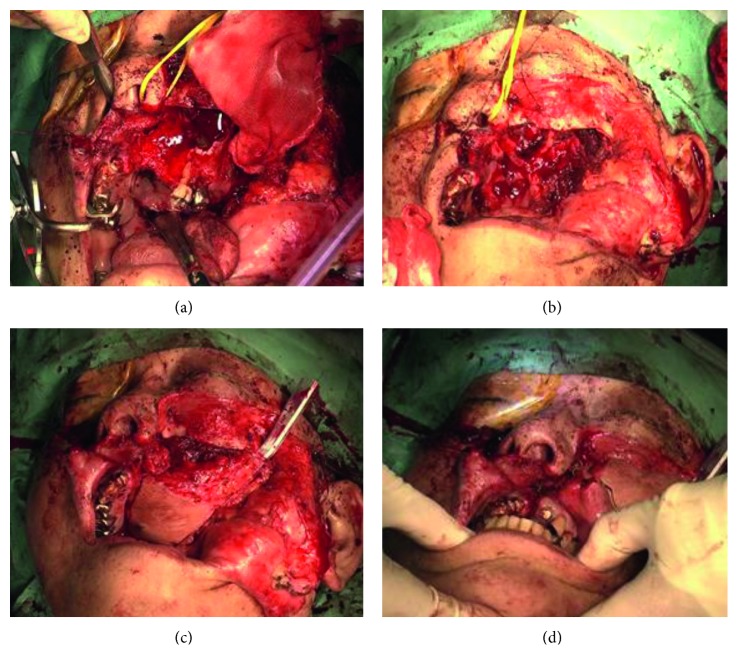
Surgical procedure of the modified partial swing approach. (a) Weber–Fergusson incision was made, and an anterior maxillary flap was elevated. (b) Tumor was excised. (c) Reconstruction was performed with an ALT flap. (d) The maxilla flap was repositioned and fixed with miniplate.

Because there was no evidence of anterior maxillary invasion, the maxillary swing approach was chosen. The surgical technique was as follows: a skin incision was made along the Weber–Fergusson incision, as in the original maxillary swing technique, and widened minimally to enable osteotomy. In order to preserve the blood supply to the maxilla, the skin was kept attached to the maxilla. An upper right incisor was missing, and bridge work had been done. The bridge was removed, and cuts were made along this line. The infraorbital nerve was sacrificed, and osteotomy was performed with a thin oscillating saw. The red lines in Figure 2 show the osteotomy lines. The resulting bone flap was then elevated (Figure 3(a)).

After the flap was elevated, the tumor outline was visualized and the posterior sections of the maxilla and pterygoid process were resected (Figure 3(b)). The cut surfaces of bone were smoothed, and a frozen section was examined for pathology and found to be negative.

Reconstruction was then performed using an anterior lateral thigh (ALT) flap (Figure 3(c)). The maxilla flap was repositioned and fixed with a miniplate (Figure 3(d)). The total operation time was 10 hours 54 minutes, and blood loss was 1200 ml.

**Figure 4:**
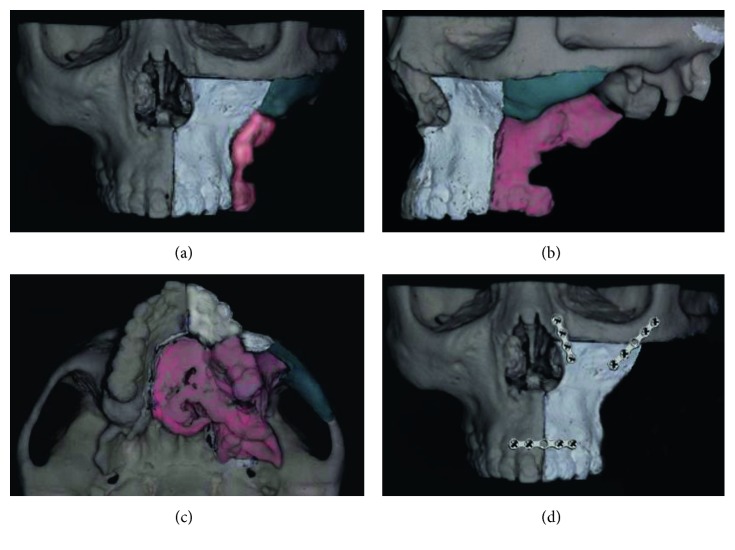
(a) Frontal view of model surgery. (b) Lateral view of model surgery. (c) Look-up view of model surgery. (d) View after tumor was resected, and the anterior maxilla flap was fixed with three miniplates.

Mock surgery was performed on a 3D printer-generated model prior to the operation (Figure 4). The lines to be cut were identified on the 3D model. The white area represents the swung anterior maxilla, and the blue area (inferior part of the zygomatic arch) represents the area removed for visualization purposes. The red area represents the dissected area. The actual operation was performed while referencing this model and using the same techniques employed during the mock surgery.

**Figure 5:**
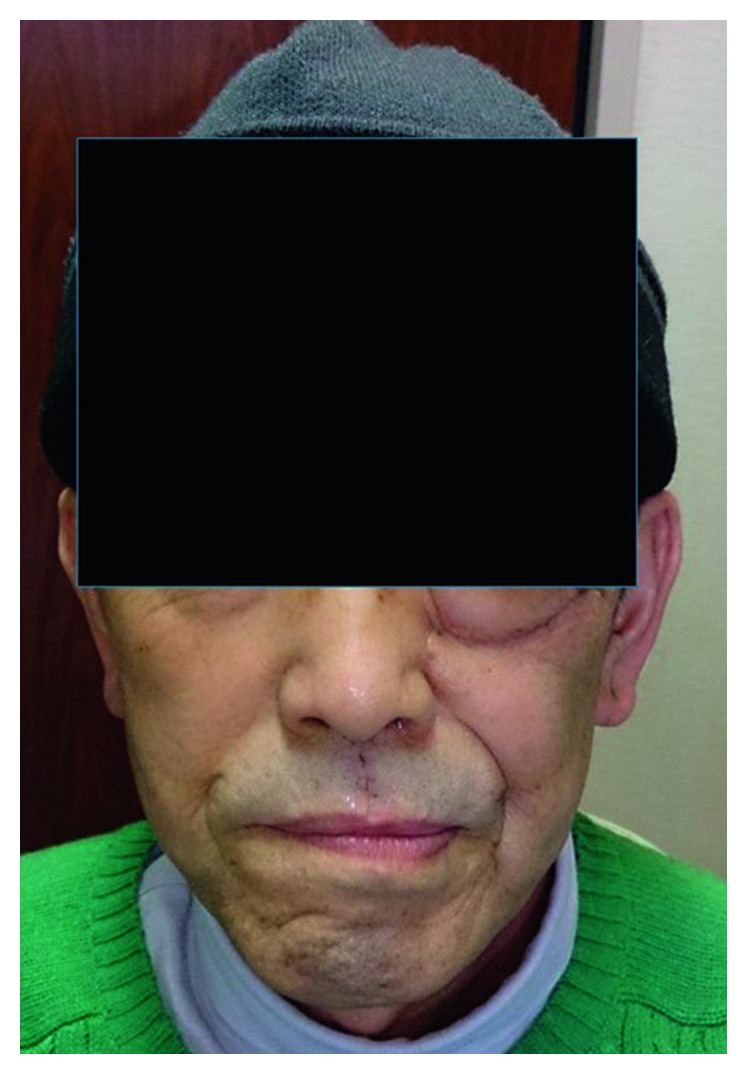
Frontal view two months after surgery.

Figure 5 shows a frontal view of the patient two months after surgery. Buccal deformity was minimal and acceptable. Five months after surgery, bilateral neck and retropharyngeal metastasis occurred, and bilateral neck dissection and postoperative radiation were performed.

## 3. Discussion

Since midface and cranial base bone areas are anatomically very complicated, adequate visualization for tumor resection is needed. Additionally, since severe complications occur if large incisions are made, the selection of the surgical approach and reconstruction are very important. For facial translocation of the cranial base, Janecka et al. [4] presented the basic concept of the approach to be used in this area.

Palatal tumors, especially those invading to the pterygoid fossa, are difficult to dissect and reconstruct after total resection. Total resection of the maxilla and pterygoid area is a good radical procedure, but it results in large defects and causes functional and esthetic problems. The LeFort I approach is not indicated for this area because the incision line overlaps the tumor.

To overcome these issues, the original MS was introduced by Wei et al. [1]. This innovative procedure yields an excellent view of the nasopharynx, but it does include a total maxillotomy. In cases such as ours, this technique is not indicated because of concerns surrounding tumor separation.

The partial MS reported by Sumi et al. [2] features a modified maxillectomy line that is more anterior to the hard palate. With this technique, retropharyngeal areas can be resected, but the palate is not preserved. Shimoda et al. [3] reported another modified MS procedure in which osteotomy is performed on the anterior wall of the maxilla and zygomatic bone. In our case, however, this was unsuitable because the palate tumor could not be resected without sacrificing the alveolar bone.

Thus, we developed our own version of the modified partial MS that was more anterior than the previous versions. We also created an anterior maxilla and alveolar ridge flap. As this area is crucial for both esthetic reasons and mastication purposes, the blood supply to the flap is a major concern. Careful setup and maintenance of the flap enabled us to preserve its blood supply.

For reconstruction, we used an ALT flap due to its many advantages (e.g., low donor site morbidity and tissue versatility). Parkes et al. [5] showed the utility of ALT in cranio-orbitofacial reconstruction, and ALT reconstruction was extremely useful in our MS approach.

In terms of functional results, postoperative mastication and swallowing functions were good. Speech function was also good, and esthetic appearance was acceptable due to our preservation of the infraorbital and alveolar frame.

## 4. Conclusion

The maxillary swing approach can be applied not only to the nasopharynx and retromaxillary area but also to palatal tumors. This technique is particularly useful for preserving appearance as well as speech and mastication.
